# The functional alterations in primary migraine: A systematic review and meta-analysis protocol

**DOI:** 10.1097/MD.0000000000019019

**Published:** 2020-03-06

**Authors:** Guixing Xu, Shirui Cheng, Yuzhu Qu, Ying Cheng, Jun Zhou, Zhengjie Li, Fanrong Liang

**Affiliations:** a The Acupuncture and Tuina School, The 3rd Teaching Hospital, Chengdu University of Traditional Chinese Medicine; b The First Affiliated Hospital of Chengdu University of Chinese Medicine, Chengdu, Sichuan, China.

**Keywords:** fMRI, functional cerebral alterations, meta-analysis, migraine, neuroimaging studies, PET, protocol, signed differential mapping, SPECT, systematic review

## Abstract

**Introduction:**

Accumulating neuroimaging studies have found abnormal cerebral activity in migraine patients. However, the findings of studies exist many differences. Hence, this protocol aims to investigate concurrence across the neuroimaging studies to verify the functional cerebral alterations based on the latest evidence.

**Methods and analysis:**

Functional neuroimaging studies comparing migraineur with healthy subjects will be searched in the 4 online databases (EMBASE, the Cochrane Library, PubMed, and Web of Science) up to June 2019. The selection of studies, quality assessment, and data extraction will be conducted by 2 independent researchers. The Anisotropic effect size version of signed differential mapping (AES-SDM) methods will be used to conduct a coordinate-based meta-analysis. The bias of publication will be confirmed via the P value of Egger test. The quality of studies will be evaluated by the Newcastle-Ottawa Scale (NOS). This study is registered with PROSPERO, number CRD42019129043.

**Results:**

This study will deepen the understanding of functional cerebral alterations of migraine.

**Conclusion:**

The study will provide clear conclusion of the functional cerebral alterations based on the latest evidence.

**Strength and limitation**

1. This study tries to characterize the altered functional brain regions of primary migraine by both qualitative and quantitative analysis.
2. The anisotropic effect size version of signed differential mapping (AES-SDM) is an effective method for neuroimaging data synthesis based on the included studies.
3. Structural changes of primary migraine have also been demonstrated in recent studies, meta-analysis of which will be further considered in the future.

## 1 Introduction

Primary migraine (PM) is characterized by one-sided pulsating head pain, photophobia, fear of sound, nausea. It affects about 12% of adults in the USA and 9% in China.^[1,2]^ Migraine reduces patients’ daily life, leads to substantial disability and heavy economic burden on patients and society.^[1,3,4]^ Repeated attacks may increase the risk of cardiovascular, cerebrovascular diseases even lead to neurological impairment in certain brain regions.^[1,4]^

At present, the pathogenesis of migraine mainly involves the following aspects: increased excitability of neurons during migraine attacks, cortical spreading depression (CSD) triggers aura,^[5]^ trigeminal nervous system activation, and abnormal function of central pain regulation system. In recent years, the rapid development of neuroimaging has enabled people to better understand the pathogenesis of migraine, especially positron emission tomography (PET) and blood oxygenation level-dependent magnetic resonance imaging (BOLD-fMRI) are increasingly used in the study of migraine. Some resting state fMRI studies show that, compared with health people, migraineurs had a significant decrease in regional homogeneity (ReHo) values in the anterior cingulated cortex (ACC),^[6,7]^ the prefrontal cortex (PFC),^[6,7]^ the orbital frontal cortex (OFC),^[6,7]^ the supplementary motor area (SMA),^[6]^ the brainstem,^[8]^ thalamus,^[8]^ the putamen,^[8]^ temporal lobe,^[8]^ temporal cortex,^[8]^ cerebellum;^[8]^ and reduced Amplitude Of Low Frequency Fluctuation (ALFF) in rostral ventromedial medulla (RVM),^[9]^ trigeminocervical complex (TCC),^[9]^ left calcarine,^[10]^ cuneus,^[10]^ and parietal gyrus.^[10]^ Another studies found that migraineurs had an significantly increased ReHo in the OFC^[8]^ and secondary somatosensory cortex;^[8]^ and increased ALFF in posterior insula,^[9]^ putamen,^[9]^ caudate,^[9]^ right hippocampus,^[10]^ parahippocampal gyrus,^[10]^ insula,^[10]^ middle temporal gyrus^[10]^ and superior temporal gyrus.^[10]^ Some PET studies found that migraine patients had significant hypometabolism in several regions known to be involved in central pain processing, such as bilateral insula,^[11]^ bilateral anterior and posterior cingulate cortex,^[11]^ left premotor and prefrontal cortex,^[11]^ left primary somatosensory cortex,^[11]^ dorsal pons's mouth,^[12]^ ACC,^[12]^ wedge-leaf renal cortical blood flow (rCBF),^[12]^ OFC,^[13]^ and rostral anterior cingulate cortices (rACC),^[13]^ compared with healthy people. Another PET study^[14]^ found that the regions of higher metabolism were large, bilateral, heterogeneous, and located mainly in the posterior white matter of the cerebrum and cerebellum, occipital, and temporal regions. A SPECT studies found decreased rCBF on both sides of the frontal^[15]^ and temporal lobes,^[15]^ left parietal lobe,^[15]^ and right occipital lobe,^[15]^ and increased rCBF focus on the left prefrontal lobe,^[15]^ and right temporal lobe.^[15]^ These studies show that the abnormal cerebral functional associated with migraine headaches, but not all data reported entirely consistent findings, and even the inconsistent results consist in some study, such as OFC,^[6–8]^ putamen,^[8,9]^ temporal region,^[8,10,14,15]^ etc. The main reasons may due to the different selection of headache diseases in the study, the small sample size, the pain site and the pain duration and frequency are different, resulting in poor comparability between results, even different studies have reached the opposite conclusion.^[16–24]^

The meta-analysis is often used to evaluate evidence accurately and objectively. The methods commonly used to perform a meta-analysis of neuroimaging data include the Anisotropic effect-size version of Seed-based d Mapping (AES-SDM), multilevel kernel density analysis (MKDA) and activation likelihood estimate (ALE). Compared to ALE and MKDA, AES-SDM has a higher sensitivity, overlap, and good control of false positives.^[25]^ Besides, the imprecision of AES-SDM is lower than other coordinate-based methods, which is more reliable and valid for neuroimaging studies.^[25]^

Therefore, this protocol aims to investigate concurrence across fMRI/SPECT/PET studies using AES-SDM software to help reveal the functional cerebral alterations underpinning this condition.

## 2 Methods and analysis

The meta-analysis that synthesizes the latest evidence of neuroimaging will be carried out via using AES-SDM, which comprehensive reviews the functional cerebral alterations of migraine in order to better understand the pathophysiology of migraine. This protocol has been registered on PROSPERO (https://www.crd.york.ac.uk/PROSPERO/). The PROSPERO registration number: CRD42019129043. The protocol is according to PRISMA-P statement guidelines.^[26]^ And the results of this meta-analysis will be published in a journal or conferences.

### 2.1 Criteria of selection for study

#### 2.1.1 Criteria for inclusion

1. Studies of comparing functional cerebral alterations of migraineur with that of healthy controls will be included.
2. Adult patients (aged ≥18 years) diagnosed with PM according to the criteria for migraine from the International Headache Society.
3. Whole-brain results in three-dimensional coordinates (x, y, z) of changes in standard stereotactic space (Talairach or MNI) were reported.
4. Thresholds for significance corrected for multiple comparisons or uncorrected with spatial extent thresholds were used.
5. The PET/ALFF/FLAFF/ReHo/SPECT data of functional cerebral alterations is used.
6. The study is available in the English language.

#### 2.1.2 Criteria for exclusion

1. Studies only reporting region of interests (ROIs) findings were excluded.
2. Studies using coordinates relative to analyze employing small volume corrections (SVC) in preselected ROIs were excluded.
3. A study with a sample size under 10 will be excluded to ensure the reliability of the outcome.

### 2.2 Search methods for identification of studies

#### 2.2.1 Electronic searches

From the inception dates to June10, 2019, the following databases will be searched: EMBASE, the Cochrane Library, PubMed, and Web of Science. The searching strategy of Cochrane library database is presented in Table 1.

**Table 1:**
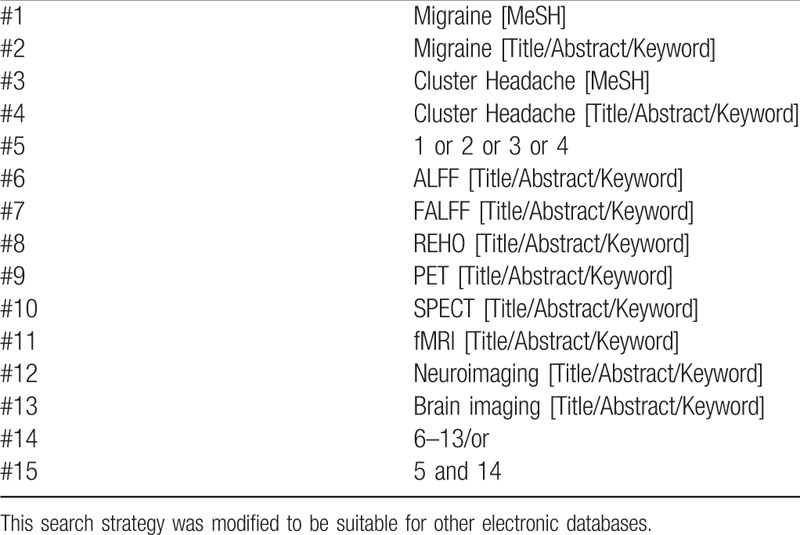
Search strategy for Cochrane library database.

#### 2.2.2 Searching other resources

Additional trials will be further identified according to the list of all identified publications including relevant systematic reviews and meta-analysis.

### 2.3 Data collection and analysis

#### 2.3.1 Selection of studies

**Figure 1:**
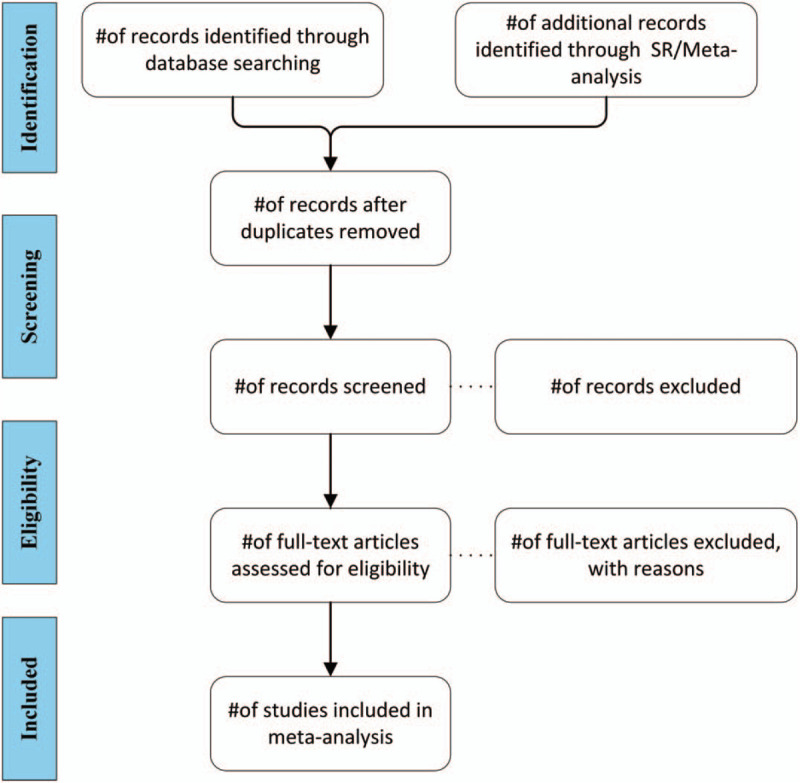
Study flow diagram, illustrate the process of studies selection.

Before the selection of publications, the study screening process will be counseled and developed among all the reviewers. After electronic searches, the results will be exported to a database called “migraine” created by Endnote software (version X9). Publications obtained from other sources will also be imported to the same database. Two reviewers (XGX and CSR) will independently screen out the titles and abstracts in this database according to the criterion below: first, find out and delete the duplicates (same content in different languages or different published forms such as journal articles and conference abstracts, or 2 articles including the same trial from different aspects); second, exclude studies which have no baseline comparison or participants are diagnosed with other severe conditions (such as severe nephropathy, heart diseases, cancer, and so on); third, control group that are not healthy participants will be excluded; fourth, studies including participants under the age of 18 will be excluded. If it is hard to screen the studies based on the titles and abstracts, the full text of these studies will be screened furtherly. When disagreements occur between the 2 reviewers, they will be resolved through discussion or the third reviewer (QYZ). The diagram of the selection of studies is shown in Figure 1. A list of all the excluded studies with reasons will be presented.

#### 2.3.2 Assessment of quality in included studies

The quality of all included studies will be assessed using NEWCASTLE-OTTAWA QUALITY ASSESSMENT SCALE checklist presented in Appendix 1,^[27]^ which focuses on subjects, comparability between groups, and measurement of exposure factors.

#### 2.3.3 Data extraction and management

Before data extraction, a standardized data extraction form will be developed by the meeting of all reviewers. Then we will use this form to extract information from at least 3 studies to check its feasibility. Two reviewers (XGX and CSR) will extract the following information from the database: general characteristics, including year of publication, reference ID, reviewer's name, the first author of the study, publication source, etc.; study characteristic, including design of trial (design of the study, PM and control group, method of analysis, etc.), participants (age, gender, ethnicity, country, diagnosis, duration, chronic or episodic, with or without aura, etc.), method of acquired data (type of neuroimaging used, power of the MRI magnetic field, etc.), results (mean, SD, coordinate, total sample size, etc.) etc..

The disagreement between the 2 reviewers will be solved by discussion among all the reviewers. The extraction data will be listed in Excel 2016, and ZJ will check the data input to ensure the consistency and correct data entry errors.

### 2.4 Meta-analysis

The units of each data from different trials will be converted to the International System of Units before statistical analysis. The *P* value and *T* value, the outcome of a comparison between HC and patients with PM, will be converted to *Z* value. The unit of age and duration will be uniform for year and month. And the author plus year will be set as the study ID.

Cerebral functional alternated brain regions will be voxel-wise meta-analyzed with the AES-SDM, which has already been used in many studies such as schizophrenia, psychosis, depressive disorders.^[28,29]^ The meta-analysis of PM will be implemented with standard random-effects variance-weighted. An uncorrected *P* < .005 is set as the main threshold, with an additional peak height *Z* > 1 and cluster extent ≥10 voxels to optimally balance the sensitivity and specificity.^[25]^ In this step, the mean analysis represents the weighted mean difference in the regional grey matter between patients with PM and healthy controls.

The *Q* statistic maps will be used to explore those brain regions with higher heterogeneity. The standard z-values, automatically converted by *Q* value in AES-SDM, is related to summarize the differences between more than 2 groups (or the effect of more than 1 regressor).^[2]^

When more than 10 studies are included, it is sufficient to detect publication bias in meta-analytical procedures.^[30,31]^ The probability threshold was decreased to 0.005 to minimize the detection of false correlation.

### 2.5 Meta-regression or subgroup analysis

If sufficient trials are included, we will explore the following potential sources of heterogeneity using subgroup analyses or meta-regression:^[25]^ the different methods of MRI/PET/SPECT measuring including scan-T and FWHM; disease classification about PM with aura or without aura; mean age of the patients; mean durations of the patients; mean frequency of patients; etc.

### 2.6 Sensitive analysis

Leave-one-out jackknife sensitivity analysis was used to test the stability of findings of the fMRI studies, which consists of repeating the mean analysis by systematically removing each study and repeating the analysis.

## 3 Results reporting and presentation

The resulting systematic review and meta-analysis will follow the Preferred Reporting Items for Systematic reviews and Meta-Analyses(PRISMA) statement.^[26]^ Flow diagram will be used for the process of study selection. Reasons for study exclusion will be described. Quantitative data will be presented in summary tables and map where appropriate. The quality scores and risk of bias for each eligible study will be reported.

## 4 Ethics and dissemination

This review does not require ethical approval duo to data that we will not endanger the individual's privacy or compromise their rights. The results of the review that provide systematically view and evidence of neuroimaging of migraine will also give implication for clinical practice understanding the physiopathology of migraine and further research, and the founding of this study may be published in a peer-reviewed journal or distributed at relevant conferences.

### 4.1 Patient and public involvement

No patient involved.

## Acknowledgments

We thank all the authors of the included studies. We especially thank Prof. Fang Zeng for her kind help and suggestions.

## Author contributions

Guixing Xu conceived the review protocol and drafted the manuscript. Shitui Cheng, and Yuzhu Qu revised the study design. Guixing Xu, Shitui Cheng, and Yuzhu Qu participated in the design of the search strategy and data extraction data set. Guixing Xu, Shitui Cheng, Fanrong Liang and Zhengjie Li formed the data synthesis and analysis plan. In practice, Jun Zhou and Ying Cheng will monitor each procedure of the review and are responsible for the quality control. All authors have read and approved the publication of the protocol.
